# Dietary Factors and Risk of Glioma in Adults: A Systematic Review and Dose-Response Meta-Analysis of Observational Studies

**DOI:** 10.3389/fnut.2022.834258

**Published:** 2022-02-14

**Authors:** Weichunbai Zhang, Jing Jiang, Xinyi Li, Yongqi He, Feng Chen, Wenbin Li

**Affiliations:** ^1^Department of Neuro-Oncology, Cancer Center, Beijing Tiantan Hospital, Capital Medical University, Beijing, China; ^2^College of Nursing, University of South Florida, Tampa, FL, United States

**Keywords:** glioma, meta-analysis, dose-response relationship, observational study, dietary factors

## Abstract

**Background:**

Gliomas are the most common primary intracranial tumors in adults. Inappropriate dietary habits are thought to be a risk factor for most human cancer, and glioma is no exception. However, the effect of dietary factors on glioma is not clear.

**Objective:**

This review aims to quantitatively evaluate the association between various dietary intakes and glioma using a meta-analysis.

**Methods:**

We searched articles on PubMed, the Cochrane Library, the Web of Science, and EMBASE from their inception until October 11, 2021. According to heterogeneity, the fixed-effects or random-effects model was selected to obtain the relative risk (RR) of merger. Based on the methods described by Greenland and Longnecker, we explored the dose-response relationship between dietary intakes and the risk of glioma. Subgroup analysis, sensitivity analysis, and publication bias were also used.

**Results:**

This study reviewed 33 articles, including 3,606,015 controls and 8,831 patients with glioma. This study included 12 food groups. Compared with the lowest intakes, the highest intakes of tea (*RR* = 0.82, 95%CI:0.71–0.93), total vegetables (*RR* = 0.84, 95%CI: 0.70–1.00), green vegetables (*RR* = 0.80, 95%CI: 0.66–0.98), and orange vegetables (*RR* = 0.79, 95%CI: 0.66–0.96) significantly reduced the risk of glioma, while the highest intakes of grains (RR = 1.39, 95%CI: 1.16–1.66), processed meats (RR = 1.19, 95%CI: 1.00–1.42), and processed fish (RR = 1.37, 95%CI: 1.03–1.84) significantly increased the risk of glioma. The results of subgroup and sensitivity analyses remained unchanged. In the dose-response relationship, only tea was statistically significant. Taking an extra cup of tea every day reduced the risk of glioma by 4%.

**Conclusions:**

Our analysis suggests that the intakes of tea, total vegetables, green vegetables, and orange vegetables may reduce the risk of glioma, while the intakes of grains, processed meats, and processed fish may increase the risk of glioma. Therefore, the effect of dietary factors on glioma should not be ignored.

**Systematic Review Registration:** https://www.crd.york.ac.uk/prospero/, CRD42022296658.

## Introduction

Gliomas are the most prevalent types of adult brain tumors, accounting for 78% of malignant brain tumors (1). Because of its low morbidity, high mortality, rapid onset, and easy recurrence, it has caused a serious disease burden for people. Due to the aggressive nature of gliomas, a complete surgical resection is difficult to achieve (2). The prevention of glioma has become one of the important anti-disease strategies. Therefore, the etiology of glioma has become a major focus in the past three decades. Several studies have focused on endogenous factors such as allergic diseases (3), genetic susceptibility, head injury (4), and multiple alleles that have been found to be associated with glioma risk, while frequent exposures to ionizing radiation have been found to significantly increase glioma risk among environmental risk factors (5).

Inappropriate dietary habits such as long-term consumption of processed meats (grilled, smoked, cured red, and white meats) and insufficient intakes of vegetables and fruits are thought to be a risk factor for most human cancer, and glioma is no exception (6). Currently, a few studies that investigate dietary-assisted glioma therapy have found mostly positive effects (7, 8). However, the results of nutritional epidemiological studies on the etiology of glioma are not satisfactory. Although a few studies have been reported on the effect of daily dietary factors, including vegetables, fruits, meats, fish, and non-alcoholic beverages, the evidence remained difficult to reconcile. Terry et al. (9) found a protective effect of higher intakes of vegetables against glioma in a multicenter case-control study [odds ratio (OR) = 0.7, 95%CI: 0.5–0.9]. And, both green and orange vegetables had independent protective effects. Chen et al. also found that the intakes of vitamin A and carotene-rich in vegetables were significantly negatively correlated with glioma in the case-control study (vitamin A: OR = 0.5, *P*_−*trend*_ = 0.005; α-carotene: OR = 0.5, *P*_−*trend*_ = 0.01, β-carotene: OR = 0.5, *P*_−*trend*_ = 0.01) (10). Blowers et al. found that nitroso-exposed diets such as processed meats increase the risk of glioma, especially bacon (OR = 6.6, 95%CI: 1.9–22.5), but taking vitamin supplements seems to have a protective effect (11). None of the observational studies on fish intakes and glioma found significant results (10–12), but a meta-analysis showed that dietary intakes of fresh fish reduced the risk of glioma [relative risk (RR) = 0.823, 95%CI: 0.70–0.97] (13). A population cohort study with an average follow-up of 14.1 years found no evidence that various types of meats (red meats, processed meats, or subtypes of meats) or iron (total or heme) was associated with glioma (14). Similar results were found for the other processed meats (15, 16). The impact of tea and coffee on glioma has also attracted much attention. Two prospective studies have shown that tea has a protective effect against glioma. Every extra cup of tea daily could reduce the risk of glioma by 7%, while the result of coffee was not significant (17, 18). Pranata et al. obtained the same conclusion through a meta-analysis (19). However, in three large prospective cohort studies in the UK and the USA, Kuan et al. analyzed the effects of 15 food groups on glioma at different follow-up times, no significant effect was found (20). Finally, the incidence rate of gliomas is significantly lower compared with other cancers. Thus, only a relatively small number of cases can be obtained even with large cohort studies. This may be the main reason for the inconsistent research results.

To provide a quantitative assessment of the effect of dietary intakes on glioma risk, we synthesized all published observational studies on dietary factors and glioma. We used the dose-response meta-analysis to quantify this association between dietary factors and glioma risk and to determine whether the relationship is linear, targeting to reach some evidence for dietary factors to prevent glioma.

## Methods

### Search Strategy

Two authors independently conducted an extensive search of the Cochrane Library, PubMed, Web of Science, and Embase until October 11, 2021. The Cochrane Library search terms used for the title, abstract, and keywords were “glioma” OR “brain cancer” OR “brain tumor” combined with “diet” OR “food” OR “lifestyle” OR “nutrition” OR “fruit” OR “vegetable” OR “meat” OR “coffee” OR “tea” OR “fish” OR “vitamin.” The same retrieval strategy was also applied to the other databases. No document type, language, or other relevant restrictions were used in the retrieval process, and the unpublished articles were excluded. Two reviewers screened the titles and abstracts to select the articles and reviewed the full text. Any disagreements between the two authors were settled by a third author. In addition, we searched the references of the published meta-analysis to identify other potential articles.

### Inclusion and Exclusion Criteria

For the meta-analysis, we included the articles that met the following criteria: (1) Exposure: the dietary intakes of participants. The exposure of interest was dietary intakes of participants. The studies gave the daily intakes of each food or the overall intakes of a food group through food frequency questionnaires or dietary recall; (2) Outcome: glioma; and (3) Population: 18 years old and above. This was due to the large dietary differences between minors and adults.

The exclusion criteria of the meta-analysis were as follows: (1) study population included minors (<18 years of age); (2) non-observational study (reviews, case reports, and clinical trials); (3) lacking effect size and 95%CI; and (4) if multiple studies used the data from the same population, a study with the largest sample size was included in this meta-analysis.

### Data Extraction

For the articles that conformed to the inclusion criteria, the data in the articles were extracted independently by two authors according to the predesigned format. The extracted data included the first author, year of publication, country, study population, study type, age, sex, sample size, number of cases, dietary intake level, effect size, and 95%CI extracted from the most adjusted model. In case of disagreement during data extraction, the conflict would be submitted to a third author for adjudication.

### Quality Assessment

Each study was evaluated by two authors and handed over to a third party for adjudication in case of disagreement. As the included articles were observational studies, the Newcastle–Ottawa scale (NOS) was used to evaluate the quality of the study and the possible risk of bias (21).

### Statistical Analysis

For the current meta-analysis, we conducted a meta-analysis based on the effect size and 95%CI between the highest quantile and the lowest or reference quantile of dietary intakes. Heterogeneity between studies was assessed by *I*^2^ statistics. If the heterogeneity was not statistically significant (*I*^2^ <50% and *p* > 0.10), the fixed-effects model was used to combine the effect size and 95%CI. Otherwise, the random-effects model was used. We conducted a subgroup analysis to determine whether the heterogeneity of the study came from the study type (case-control study and cohort study), the study population (European population, American population, etc.), and the study quality (>7 points, ≤ 7 points), to explore the potential sources of heterogeneity. We performed a sensitivity analysis. We successively omitted one study at a time to assess each study's relative impact on the total effect size estimation. Different statistical models (fixed-effects and random-effects model) were used to estimate effect size. Egger's test and Begg's test were used to detect publication bias.

Subsequently, for dietary factors with significant results in the analysis of extreme categories, we also explored the dose-response relationship between dietary intakes and glioma risk. The method developed by Greenland and Longnecker was used to analyze the dose-response relationship in this study (22). For this method, we need to extract at least three groups of dietary intakes, number of participants, number of cases, effect size, and 95%CI for each type of dietary factors in each study. For differences of measurement used in different studies, we calculated and unified them into cups (coffee and tea, 1 cup = 8 ounce) or grams (other dietary factors). For each study, the median or average dietary intake corresponding to each group was used for risk estimation. If the median or average dietary intake of each group was not provided, the midpoint of the upper and lower limits of each group should be designated as the average exposure level. If the highest group was open, we assumed that the interval width was the same as the second highest category. *Q*-value was applied to assess between-study heterogeneity.

Stata 14.0 was used for all statistical analyses. Unless otherwise noted, *p* < 0.05 was considered as statistically significant.

## Results

### Study Characteristics

**Figure 1:**
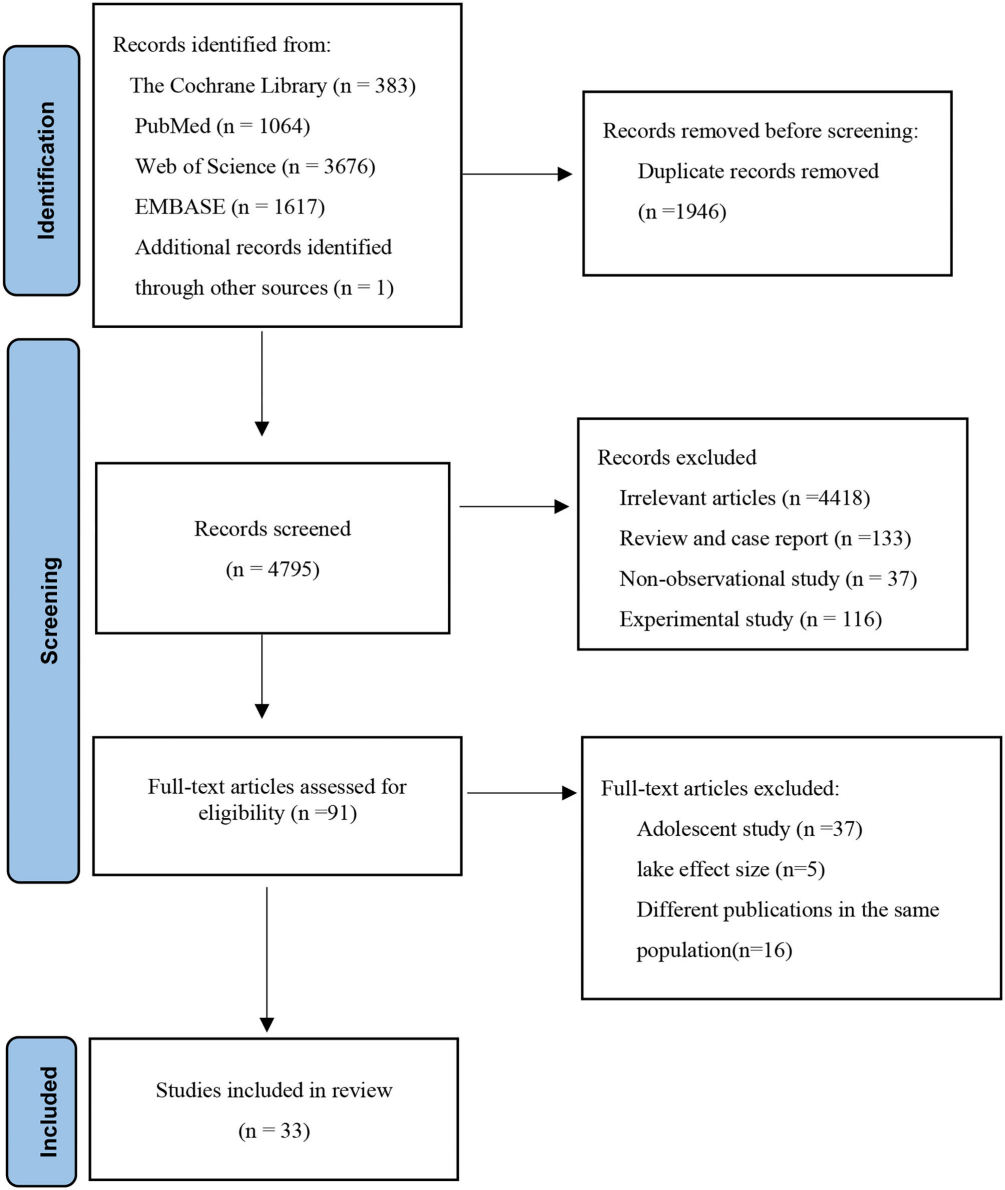
A flow diagram outlining the systematic search and article selection process.

Figure 1 shows the article screening process of this study. A total of 6,741 articles were retrieved, including 383 from the Cochrane Library, 1,064 from the PubMed, 3,676 from the Web of Science, 1,617 from EMBASE, and 1 from other sources. After excluding duplicates between different databases, titles and abstracts of 4,795 articles were reviewed. A total of 4,704 articles were excluded because they were not related to the aim of this study. Non-observational studies and animal/cell experiments or reviews were excluded. Then, 91 articles were reviewed in full text, and 58 articles were excluded due to adolescent research, lacking effect size, and the duplication of the study population. A total of 33 articles were included.

Table 1 presents a summary of the 33 articles and characteristics included in this meta-analysis (9–12, 14, 15, 17, 18, 23–47). All studies included 3,606,015 controls and 8,831 patients with glioma. Most participants were 18–80 years old, and each study included two sex groups. The included studies were mainly concentrated in America and Europe (Britain, France, Germany, and Sweden), and a few studies were completed by Canada, Australia, China, Japan, and Iran, including 18 case-control studies and 15 cohort studies. These studies provide glioma-related results for a total of 12 food groups: coffee, tea (black tea, green tea, and herbal tea), total vegetables, green vegetables (broccoli and green leafy vegetables such as spinach, silver beet, and lettuce), orange vegetables (pumpkin, carrot, tomato, etc.), grains, processed meats (grilled, smoked, cured red, and white meats such as bacon, sausage, luncheon meats, cold cuts, smoked ham, and hot dogs), red meats, fresh fish, processed fish (salt fish, smoked fish, pickled fish), total fruits, and citrus fruits. Around 50% of the studies had a NOS score of 8 or more.

**Table 1:** Characteristics of studies investigating the association between dietary factors and glioma.

**References**	**Year**	**Country**	**Study type**	**Age**	**Sample size**	**Cases**	**Dietary factors**	**Effect size (95%CI)**	**Quality score**
Ahlbom et al. (23)	1986	Sweden	Case-control	20–75	275	78	Processed meats	1.81 (1.16–2.83)	8
							Processed fish	0.90 (0.30–2.50)	
Burch et al. (24)	1987	Canada	Case-control	25–80	360	180	Coffee	1.40 (0.76–2.58)	7
							Tea	1.26 (0.70–2.25)	
							Processed meats	0.50 (0.23–1.07)	
							Processed fish	1.67 (0.61–4.59)	
							Total fruits	0.35 (0.18–0.71)	
							Citrus fruits	0.56 (0.32–0.98)	
Mills et al. (25)	1989	America	Cohort	>25	34,000	20	Processed meats	2.29 (0.51–7.77)	8
							Total fruits	0.85 (0.28–2.60)	
							Citrus fruits	0.92 (0.37–2.37)	
Hochberg et al. (15)	1990	America	Case-control	18–81	288	160	Coffee	0.90 (0.50–1.80)	7
							Processed meats	1.00 (0.60–1.60)	
Preston-Martin et al. (26)	1991	America	Case-control	25–69	404	202	Processed meats	1.00 (0.20–4.20)	6
Boeing et al. (27)	1993	Germany	Case-control	25–75	520	105	Total vegetables	0.90 (0.50–1.70)	7
							Processed meats	2.10 (1.10–4.00)	
							Processed fish	1.40 (0.80–2.40)	
							Total fruits	1.10 (0.60–1.90)	
Gile et al. (12)	1994	Australia	Case-control	20–70	818	409	Total vegetables	0.75 (0.40–1.41)	7
							Green vegetables	0.86 (0.61–1.22)	
							Orange vegetables	0.94 (0.65–1.35)	
							Processed meats	1.31 (0.92–1.86)	
							Grains	1.17 (0.68–2.01)	
							Fresh fish	0.94 (0.47–1.89)	
							Processed fish	1.31 (0.84–2.06)	
							Total fruits	1.06 (0.49–2.27)	
Blowers et al. (11)	1997	America	Case-control	25–74	188	94	Total vegetables	1.30 (0.50–3.20)	7
							Processed meats	1.70 (0.80–3.80)	
							Grains	2.80 (1.20–6.50)	
							Fresh fish	0.40 (0.20–1.10)	
							Processed fish	1.70 (0.80–3.80)	
							Total fruits	1.30 (0.50–3.00)	
							Citrus fruits	1.70 (0.70–4.30)	
Lee et al. (28)	1997	America	Case-control	>20	866	428	Processed meats	1.76 (1.18–2.64)	8
Hu et al. (29)	1998	China	Case-control	39.6	654	218	Total vegetables	0.51 (0.29–0.89)	6
							Total fruits	0.28 (0.16–0.51)	
Schwartzbaum et al. (30)	1999	America	Case-control	44.9	80	40	Processed meats	2.30 (0.80–6.70)	7
Xu et al. (31)	1999	China	Case-control	≥18	258	86	Total vegetables	0.81 (0.73–0.89)	7
							Total fruits	0.70 (0.54–0.91)	
Chen et al. (10)	2002	America	Case-control	≥21	685	236	Total vegetables	0.50 (0.30–1.00)	7
							Green vegetables	0.70 (0.40–1.20)	
							Orange vegetables	0.60 (0.30–1.00)	
							Processed meats	1.10 (0.60–2.10)	
							Grains	1.50 (0.90–2.50)	
							Red meats	0.90 (0.50–1.60)	
							Citrus fruits	1.00 (0.60–1.70)	
Efird et al. (32)	2004	America	Cohort	>25	1,29,393	122	Coffee	1.70 (0.80–3.60)	7
Rollison et al. (33)	2004	America	Case-control		43	15	Processed meats	3.72 (0.51–27.16)	6
Holick et al. (34)	2007	America	Cohort	25–75	2,29,638	296	Total vegetables	1.17 (0.78–1.75)	8
							Orange vegetables	0.91 (0.61–1.35)	
							Total fruits	1.41 (0.95–2.10)	
							Citrus fruits	1.40 (0.93–2.13)	
Michaud et al. (35)	2009	America	Cohort	25–75	2,30,655	335	Processed meats	0.92 (0.48–1.77)	8
							Red meats	1.09 (0.62–1.93)	
Terry et al. (9)	2009	America	Case-control	20–80	3,671	1,185	Total vegetables	0.70 (0.50–0.90)	7
							Green vegetables	0.80 (0.60–1.00)	
							Orange vegetables	0.70 (0.50–0.90)	
							Processed meats	0.90 (0.70–1.20)	
							Red meats	1.30 (1.00–1.70)	
							Grains	1.30 (1.10–1.70)	
							Fresh fish	0.90 (0.70–1.10)	
							Citrus fruits	1.40 (1.10–1.80)	
Michaud et al. (36)	2010	Britain	Cohort	20–70	4,10,970	343	Coffee	0.98 (0.67–1.41)	9
							Tea	1.05 (0.75–1.48)	
Dubrow et al. (37)	2010	America	Cohort	50–71	5,45,770	585	Total vegetables	1.17 (0.89–1.53)	7
							Processed meats	1.05 (0.80–1.37)	
							Red meats	0.85 (0.65–1.11)	
							Total fruits	1.16 (0.89–1.52)	
Holick et al. (38)	2010	America	Cohort	25–75	2,19,515	335	Coffee	0.80 (0.54–1.17)	8
							Tea	0.71 (0.45–1.12)	
Baglietto et al. (39)	2011	Australia	Cohort	27–81	39766	67	Coffee	0.51 (0.23–1.10)	8
Cabaniols et al. (40)	2011	France	Case–control	≥18	244	122	Total fruits	0.85 (0.49–1.47)	7
Dubrow et al. (41)	2012	America	Cohort	50–71	5,43,006	901	Coffee	0.95 (0.64–1.41)	7
							Tea	0.75 (0.57–0.99)	
Nelson et al. (42)	2012	America	Cohort	45–68	8,006	9	Coffee	0.89 (0.08–10.02)	8
							Tea	1.21 (0.22–6.76)	
Shayanfar et al. (43)	2013	Iran	Case-control	20–75	384	128	Processed meats	0.54 (0.25–1.14)	7
							Red meats	2.50 (0.85–5.45)	
Hashibe et al. (44)	2015	America	Cohort	55–74	97,334	103	Coffee	0.76 (0.50–1.17)	8
							Tea	1.04 (0.65–1.66)	
Ogawa et al. (45)	2016	Japan	Cohort	40–69	1,01,984	61	Coffee	0.55 (0.17–1.84)	8
							Tea	1.05 (0.54–2.05)	
Ward et al. (14)	2018	Britain	Cohort	25–70	4,09,248	688	Processed meats	1.12 (0.83–1.51)	7
							Red meats	0.99 (0.75–1.31)	
Malmir et al. (46)	2019	Iran	Case-control	20–75	384	128	Coffee	0.09 (0.03–0.24)	8
							Tea	0.33 (0.13–0.86)	
Cote et al. (18)	2020	America	Cohort	25–75	2,37,516	554	Coffee	0.96 (0.66–1.37)	8
							Tea	0.73 (0.49–1.10)	
Creed et al. (17)	2020	Britain	Cohort	40–69	3,67,539	470	Coffee	0.71 (0.49–1.05)	8
							Tea	0.69 (0.51–0.94)	
Shahrestani et al. (47)	2021	Iran	Case–control	20–75	384	128	Grains	2.46 (1.01–5.97)	8

*Tea mainly included black tea, green tea, and herbal tea; Green vegetables mainly included broccoli and green leafy vegetables such as spinach, silver beet, and lettuce; Orange vegetables mainly included pumpkin, carrot, tomato, etc.; Processed meats mainly included grilled, smoked, cured red and white meats such as sausage, luncheon meats, cold cuts, smoked ham, and hot dogs; Processed fish mainly included salt fish, smoked fish, and pickled fish*.

### Effect Size Estimations of the Risk for the Association Between Dietary Factors and Glioma

Effect size estimations between all dietary intakes and the risk of glioma are presented in Table 2. Compared with the lowest intakes, the highest intakes of tea (RR = 0.82, 95%CI: 0.71–0.93, *I*^2^= 23.2%, *P*_*forheterogeneity*_ = 0.230) (Supplementary Figure 1), total vegetables (RR = 0.84, 95%CI: 0.70–1.00, *I*^2^= 53.4%, *P*_*forheterogeneity*_ = 0.029) (Supplementary Figure 2), green vegetables (RR = 0.80, 95%CI: 0.66–0.98, *I*^2^= 0, *P*_*forheterogeneity*_= 0.823) (Supplementary Figure 3), and orange vegetables (RR = 0.79, 95%CI: 0.66–0.96, *I*^2^= 0, *P*_*forheterogeneity*_= 0.422) (Supplementary Figure 4) significantly reduced the risk of glioma, while the highest intakes of grains (*RR* = 1.39, 95%CI: 1.16–1.66, *I*^2^= 21.0, *P*_*forheterogeneity*_= 0.281) (Supplementary Figure 5), processed meats (*RR* = 1.19, 95%CI: 1.00–1.42, *I*^2^= 46.4, *P*_*forheterogeneity*_= 0.019) (Supplementary Figure 6), and processed fish (*RR* = 1.37, 95%CI: 1.03–1.84, *I*^2^= 0, *P*_*forheterogeneity*_= 0.896) (Supplementary Figure 7) significantly increased the risk of glioma. However, the results of coffee, red meats, total fruits, citrus fruits, and fresh fish showed that they were not related to the incidence of glioma (Supplementary Figures 8–12).

**Table 2:** A meta-analysis for the association between dietary factors and glioma.

**Dietary factors**	**Number of studies**	**RR (95%CI)**	***I*** **^2^(%)**	***P*** **_forheterogeneity_**
Coffee	12	0.81 (0.62–1.06)	61.2	0.003
Tea	10	0.82 (0.71–0.93)	23.2	0.230
Total vegetables	9	0.84 (0.70–1.00)	53.4	0.029
Green vegetables	3	0.80 (0.66–0.98)	0	0.823
Orange vegetables	4	0.79 (0.66–0.96)	0	0.422
Processed meats	17	1.19 (1.00–1.42)	46.4	0.019
Red meats	6	1.05 (0.91–1.21)	42.5	0.122
Grains	5	1.39 (1.16–1.66)	21.0	0.281
Fresh fish	3	0.86 (0.70–1.06)	39.7	0.190
Processed fish	5	1.37 (1.03–1.84)	0	0.896
Total fruits	10	0.82 (0.59–1.12)	75.0	<0.001
Citrus fruits	6	1.12 (0.83–1.52)	52.3	0.063

### Subgroup Analysis

For the study type, tea was statistically significant in a cohort subgroup (*RR* = 0.81, 95%CI: 0.71–0.93). Total vegetables were statistically significant in the case-control subgroup (*RR* = 0.76, 95%CI: 0.67–0.87). The heterogeneity of total vegetables in the subgroup analysis of study type decreased from 53.4 to 11.0%. For the study population, tea was statistically significant in the American population (*RR* = 0.82, 95%CI: 0.68–0.98), total vegetables were statistically significant in the European population (*RR* = 0.73, 95%CI: 0.56–0.96) and other populations (*RR* = 0.76, 95%CI: 0.61–0.94). Grains were statistically significant in the American population (*RR* = 1.77, 95%CI: 1.15–2.74) and European population (RR = 1.30, 95%CI: 1.05–1.62). For study quality, tea and processed meats were statistically significant in the subgroup with the study quality score >7 (tea: *RR* = 0.81, 95%CI: 0.69–0.95; processed meats: *RR* = 1.49, 95%CI: 1.19–1.87). Total vegetables and grains were statistically significant in the subgroup with the study quality score ≤ 7 (total vegetables: *RR* = 0.82, 95%CI: 0.75–0.89; grains: *RR* = 1.38, 95%CI: 1.14–1.68). The heterogeneity of processed meats in the subgroup analysis of study quality decreased from 46.4 to 37.4% (Table 3).

**Table 3:** A subgroup analysis for the association between dietary factors and glioma.

**Dietary factors**	**Subgroup**	**Number**	**RR (95%CI)**	***I*** **^2^(%)**	***P*** **_forheterogeneity_**
Tea	Study type				
	Case-control	2	0.87 (0.53–1.43)	82.1	0.018
	Cohort	8	0.81 (0.71–0.93)	0	0.534
	Study population				
	American population	6	0.82 (0.68–0.98)	0	0.498
	European population	2	0.83 (0.66–1.05)	69.1	0.072
	Other populations	2	0.71 (0.41–1.23)	74.0	0.050
	Study quality				
	≤ 7	2	0.82 (0.64–1.06)	59.7	0.115
	>7	8	0.81 (0.69–0.95)	24.1	0.237
Total vegetables	Study type				
	Case-control	7	0.76 (0.67–0.87)	11.0	0.345
	Cohort	2	1.17 (0.93–1.47)	0	1.000
	Study population				
	American population	4	0.99 (0.69–1.43)	56.6	0.075
	European population	2	0.73 (0.56–0.96)	0	0.468
	Other populations	3	0.76 (0.61–0.94)	22.3	0.276
	Study quality				
	≤ 7	7	0.82 (0.75–0.89)	57.7	0.028
	>7	2	1.03 (0.73–1.44)	26.3	0.244
Processed meats	Study type				
	Case-control	13	1.24 (0.97–1.58)	56.5	0.006
	Cohort	4	1.08 (0.90–1.31)	0	0.682
	Study population				
	American population	11	1.19 (0.94–1.52)	33.1	0.134
	European population	4	1.30 (0.91–1.86)	71.5	0.015
	Other populations	2	0.90 (0.38–2.12)	76.8	0.038
	Study quality				
	≤ 7	12	1.07 (0.87–1.30)	37.4	0.092
	>7	5	1.49 (1.19–1.87)	8.7	0.357
Grains	Study population				
	American population	2	1.77 (1.15–2.74)	34.9	0.215
	European population	1	1.30 (1.05–1.62)	-	-
	Other populations	2	1.43 (0.90–2.27)	49.0	0.162
	Study quality				
	≤ 7	3	1.38 (1.14–1.68)	35.2	0.214
	>7	2	1.43 (0.90–2.27)	49.0	0.162
Processed fish	Study population				
	American population	2	1.69 (0.91–3.13)	0	0.978
	European population	2	1.28 (0.78–2.08)	0	0.468
	Other populations	1	1.31 (0.84–2.05)	-	-
	Study quality				
	≤ 7	1	1.70 (0.78–3.71)	-	-
	>7	4	1.33 (0.97–1.81)	0	0.860

### Sensitivity Analysis and Publication Bias

The results of the sensitivity analysis showed that the significance of the fixed-effects and random-effects model were basically the same for tea, total vegetables, green vegetables, orange vegetables, grains, processed meats, and processed fish. This suggests that the results of this meta-analysis are relatively stable (Table 4).

**Table 4:** Sensitivity analysis and publication bias.

**Dietary factors**	**Fixed-effects model**	**Random-effects model**	**Influential analysis**	**Egger's test**	**Begg's test**
Coffee	0.84 (0.74–0.99)	0.81 (0.62–1.06)	0.58–1.11	0.319	0.304
Tea	0.82 (0.71–0.93)	0.82 (0.70–1.00)	0.67–0.99	0.780	0.858
Total vegetables	0.83 (0.76–0.90)	0.84 (0.70–1.00)	0.64–1.08	0.969	1.000
Red meats	1.05 (0.91–1.21)	1.06 (0.86–1.31)	0.81–1.36	0.466	1.000
Processed meats	1.16 (1.03–1.30)	1.19 (1.00–1.42)	0.96–1.49	0.373	0.537
Grains	1.39 (1.16–1.66)	1.45 (1.14–1.85)	1.12–2.20	0.136	0.221
Fresh fish	0.86 (0.70–1.06)	0.79 (0.53–1.18)	0.39–1.15	0.531	0.296
Processed fish	1.37 (1.03–1.84)	1.37 (1.03–1.84)	0.97–2.08	0.997	1.000
Total fruits	0.87 (0.76–1.00)	0.82 (0.59–1.12)	0.54–1.21	0.607	0.858
Citrus fruits	1.22 (1.02–1.45)	1.12 (0.83–1.52)	0.70–1.64	0.377	0.452
Green vegetables	0.80 (0.66–0.98)	0.80 (0.66–0.98)	0.60–1.09	0.635	1.000
Orange vegetables	0.79 (0.66–0.96)	0.79 (0.66–0.96)	0.60–1.10	0.873	1.000

The impact of the individual study on the summary RR was assessed by repeating the meta-analysis after removing each study in turn (Table 4). For tea and processed meats, when Burch's study (24) was excluded, the results of all studies and glioma risk remained significant, but the heterogeneity decreased significantly (tea: *RR* = 0.80, 95%CI: 0.69–0.91, *I*^2^= 15.5%, *P*_*forheterogeneity*_= 0.305, processed meats: *RR* = 1.23, 95%CI: 1.04–1.46, *I*^2^= 40.4%, *P*_*forheterogeneity*_= 0.048). Similarly, excluding one grain study (11), the overall results on glioma risk were still significant, and the heterogeneity was significantly reduced (*RR* = 1.34, 95%CI: 1.12–1.62, *I*^2^= 0, *P*_*forheterogeneity*_= 0.513). It was speculated that these two studies might be the main reason for the heterogeneity of related dietary factors and glioma risk results.

Publication bias was evaluated by Begg's rank correlation method and Egger's regression test. The *p*-value of publication bias of all dietary intakes was >0.1, suggesting that the difference was not statistically significant, so the publication bias was not obvious in this study (Table 4).

### Dose-Response Relationship

**Figure 2:**
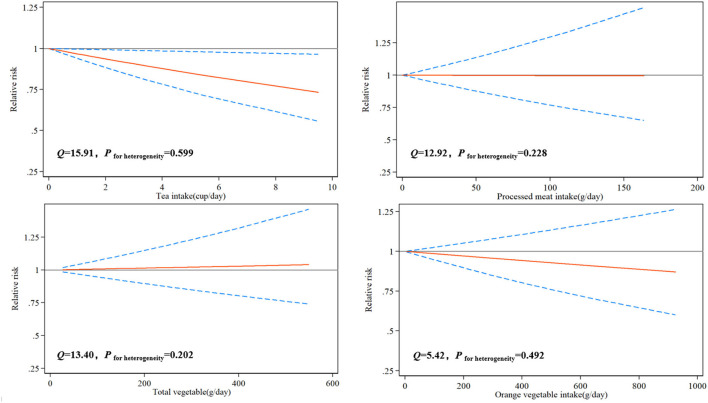
Risk between dietary factors and glioma estimates from the dose-response meta-analysis.

Due to the limited number of available articles, only tea, total vegetables, orange vegetables, and processed meats could be analyzed for the dose-response relationship from 10 articles. The dose-response relationship between various dietary factors and the risk of glioma was shown in Figure 2. There was a significant linear dose-response relationship between tea and glioma, and increasing the intake of a cup of tea every day reduced the risk of glioma by 4% (*P*_−*nonlinearity*_= 0.166, RR = 0.96, 95%CI: 0.94–0.99). Although total vegetables, orange vegetables, and processed meats had similar linear trends, the results were not significant due to an insufficient number of studies.

## Discussion

Based on 33 observational studies on dietary factors and glioma published from 1986 to 2021, involving 3,606,015 controls and 8,831 patients, our meta-analysis results showed that higher intakes of tea, total vegetables, green vegetables, and orange vegetables could significantly reduce the risk of glioma, while higher intakes of processed meats, grains, and processed fish could significantly increase the risk of glioma. However, coffee, red meats, fresh fish, fruits, and citrus fruits had no significant effect on the risk of glioma. For the results of the dose-response relationship, increasing the intake of a cup of tea per day could reduce the risk of glioma by 4%. Although there was a similar linear trend between total vegetables, orange vegetables, and processed meats and the risk of glioma, and the results were not significant. This may be due to the availability of a limited number of articles that investigate the dose-response relationship of these dietary factors on glioma.

We conducted a subgroup analysis of dietary factors with significant results due to the study population, study type, and study quality to explore the sources of heterogeneity. Although the overall heterogeneity of vegetables and gliomas was not high, it was found through a subgroup analysis that the difference of study types may be the main source of heterogeneity. Seven of the nine studies were case-control studies. Recall bias resulting from dietary survey methods in the case-control studies must be considered. There were only two cohort studies, which limit the significance of the combined results to a certain extent. The heterogeneity of processed meats mainly came from the difference in the study quality. The articles with a score of <7 still had 34.7% heterogeneity after merging. In addition, the exploration of influencing factors of glioma in the Burch study focused more on environmental exposures, such as smoking, water source, and x-ray exposure. It did not adopt appropriate investigation methods to systematically investigate the dietary intakes of the subjects but only provided a small number of dietary data such as tea and processed meats. Moreover, the cases in this study were not newly diagnosed, and the recall bias was large. These factors may explain the heterogeneity in the studies involving tea and processed meats. Similarly, Blowers et al. also contributed most of the heterogeneity to the meta-analysis of cereals and gliomas. The study had a small sample size of only 94 cases. The heterogeneity of green vegetables, orange vegetables, and processed fish was very small. Because the number of studies was small, there was no subgroup analysis. In the sensitivity analysis, the dietary factors with significant results were consistent in the two models. No publication bias was found in all studies, which suggested that the research results were relatively stable.

Creed et al. found that drinking 4 cups or more of tea each day was associated with a reduction of the risk of glioma [hazard ratio (HR) = 0.69, 95%CI: 0.51–0.94] and had the same effect on glioblastoma (HR = 0.93 per 1 cup/day increment; 95%CI: 0.89–0.98), which was consistent with our results (17). For a long time, a few studies demonstrated that polyphenols in tea prevented cancer by enhancing cell antioxidant capacity and regulating epigenetic aberrations in DNA methylation, histone modification, and microRNA formation (48). Epigallocatechin gallate has especially attracted attention. A few studies showed that epigallocatechin gallate could induce cell death, prevent cell proliferation, and limit the invasion of a variety of glioma cell lines (49). Experimental results in mice also showed that epigallocatechin gallate could enhance the therapeutic effect of temozolomide on glioma (50). Similarly, the protective effect of vegetables against glioma may be related to antioxidant effects. Chen et al.'s study found that the consumption frequency per week of orange vegetables and green vegetables was 0.1 and 0.09 times higher in the control group than that in patients with glioma (10). Vegetables contain many antioxidants such as vitamins, isothiocyanates, glucosinolates, and dietary fibers. Compared with other vegetables, dark green leafy vegetables and orange vegetables are high in the carotenoids we turn into vitamin A (51). Carotenoids are also antioxidants. A meta-analysis of seven articles showed that the highest category of dietary vitamin A was significantly associated with the reduced risk of glioma (*RR* = 0.80, 95%CI: 0.62–0.98) (52). These antioxidants can activate methylation-silenced genes in cancer cells, such as O6 methylguanine DNA methyltransferase (MGMT). The gene polymorphism of MGMT is associated with the risk of glioma, whereas increased MGMT activation is thought to prevent glioma progression (36, 53). In addition, the meta-analysis by Micek et al. found a significant negative correlation between dietary intakes of phytoestrogens (such as isoflavones and lignans) and the recurrence of cancer through a meta-analysis. In patients with grade III glioma, higher dietary intakes of lignans were associated with a better cancer survival rate (*HR* = 0.48, 95%CI: 0.25–0.92) (54). So far, however, there was no prospective study to prove that vegetables can reduce the risk of glioma. There are a few studies on specific vegetables and the risk of glioma. Moreover, no similar results were found in the correlation analysis between fruits and glioma. The specific reasons need to be further explored. In future studies, it is necessary to obtain more detailed vegetable and fruit consumption history through dietary investigation, and to conduct more accurate research on the potential protective effect of specific vegetables and fruits against gliomas. Compared with red meats and fresh fish, processed meats and processed fish have a more negative effect on glioma. Processed meats and fish carry more carcinogens during processing and preservation, such as heterocyclic amines, polycyclic aromatic hydrocarbons, and N-nitroso compounds (55). Animal experiments have confirmed that N-nitroso compounds can induce glioma by reducing the repair efficiency after DNA damage (56, 57). Exposures to N-nitroso compounds during the prenatal period can result in an offspring with brain tumors (58). However, no significant association was found in prospective studies on N-nitroso compounds and gliomas (nitrate: *RR* = 1.02; 95%CI: 0.66, 1.58; nitrite: *RR* = 1.26; 95%CI: 0.89, 1.79) (34). However, we must consider that the source of N-nitroso compounds is not limited to processed food, but also drinking water, smoking, and occupational exposure. Exploring the correlation between the exposure level of related compounds in the human body and the risk of glioma may effectively solve this problem. Furthermore, processed meats and fish have increased levels of saturated fatty acids and decreased levels of the beneficial unsaturated fatty acids (59). Some studies have shown that the growth and diffusion of glioma are related to fatty acid metabolism *in vivo* (60), and unsaturated fatty acids have a certain cytotoxic effect on glioma cells (61). Epidemiological studies show that dietary intakes of polyunsaturated fatty acids are negatively correlated with the risk of glioma (*OR* = 0.20; 95%CI: 0.05–0.84) (62). This is the first meta-analysis to conclude that grains are a risk factor for glioma. Higher intakes of grains may increase the levels of insulin and insulin-like growth factor I (IGF-1). Some articles have shown that the IGF-1 signaling system can promote tumor progression by preventing apoptosis and stimulating tumor cell proliferation, which may be one of the main mechanisms to increase the risk of glioma (63). Moreover, long-term intakes of grains will increase the levels of oxidative stress and inflammation, and to a certain extent promote the occurrence of tumors (64, 65). Although the preventive effect of coffee on glioma has been concerned by scholars, its chlorogenic acid (66), caffeine (67), and other components have been shown to have an inhibitory effect on glioma. However, this study did not find that coffee can reduce the risk of glioma, which is consistent with previous studies (17, 18). Due to the wide processing technology and a variety of coffees, there are great differences in the active ingredients contained in different coffees (68), which will affect the evaluation of the actual intakes of anticancer ingredients such as caffeine. Therefore, the evaluation of the relationship between coffee intakes and glioma may be limited. Finally, the impact of dietary intakes on glioma may not be caused by a single food but rather a dietary pattern. Mousavi et al. found that the Mediterranean diet had a protective effect against glioma in the Iranian population (69), and similar results were also found in a cohort study (20). However, there are still a few glioma-related studies that can evaluate the whole dietary quality.

To date, our study is the largest meta-analysis with the largest sample size that investigates dietary factors and glioma. This study has some advantages. First, this study is the first meta-analysis involving the impact of multiple dietary factors on the incidence of glioma, including 12 food groups. It is the first study to find the impact of grains, green vegetables, and orange vegetables on the incidence risk of glioma through a meta-analysis. Although the number of articles is relatively small, it provides a new scientific basis for the prevention of glioma. Secondly, the included articles were strictly screened. We rejected some studies that had been included in previous meta-analyses but involved meningiomas and other brain tumors. The source of heterogeneity is thoroughly analyzed for the significant results. All these improve the accuracy of the research results. However, this study has some limitations. First, the articles used have the publication dates that span a larger timeframe. Some long-standing articles and case-control studies have problems with quality, such as large recall bias and inaccurate dietary survey methods. These will inevitably impact the analysis. Additionally, as a meta-analysis of observational studies, we may not be able to control all potential confounding factors. These confounding factors may lead to a certain level of deviation. Finally, for the dose-response relationship, only tea obtained significant results. Other dietary factors did not obtain meaningful results due to a small number of articles that were included in the analysis. In future research, we can improve the relevant analysis by supplementing more articles.

## Conclusion

In summary, the current meta-analysis shows that the intakes of tea, total vegetables, green vegetables, and orange vegetables may reduce the risk of glioma, while the intakes of grains, processed meats, and processed fish may increase the risk of glioma. Therefore, the impact of dietary factors on glioma could not be ignored. In future studies, we should find a method to comprehensively evaluate dietary factors and nutrient exposure in the population, to further study the relationship between dietary factors and glioma.

## Data Availability Statement

The original contributions presented in the study are included in the article/Supplementary Material, further inquiries can be directed to the corresponding author.

## Author Contributions

WL and WZ contributed to the conception or design of the work. WZ and JJ contributed to searching the databases. WZ, JJ, and YH contributed to the acquisition, analysis, or interpretation of data for the work. WZ and XL performed proofreading and modified the language. XL and FC reviewed and edited the manuscript. All authors have read and approved the final manuscript.

## Funding

This study was supported by the National Natural Science Foundation of Beijing (No. J200003) and National Science and Technology Major Project of China (No. 2016ZX09101017).

## Conflict of Interest

The authors declare that the research was conducted in the absence of any commercial or financial relationships that could be construed as a potential conflict of interest.

## Publisher's Note

All claims expressed in this article are solely those of the authors and do not necessarily represent those of their affiliated organizations, or those of the publisher, the editors and the reviewers. Any product that may be evaluated in this article, or claim that may be made by its manufacturer, is not guaranteed or endorsed by the publisher.
