# Supplementary material for: Dietary Factors and Risk of Glioma in Adults: A Systematic Review and Dose-Response Meta-Analysis of Observational Studies
Source: Front Nutr. 2022 Feb 14;9:834258. doi: 10.3389/fnut.2022.834258 (PMC8883057; doi:10.3389/fnut.2022.834258)
Supplement: Supplementary file 1 [file Data_Sheet_1.docx]

Supplementary Material
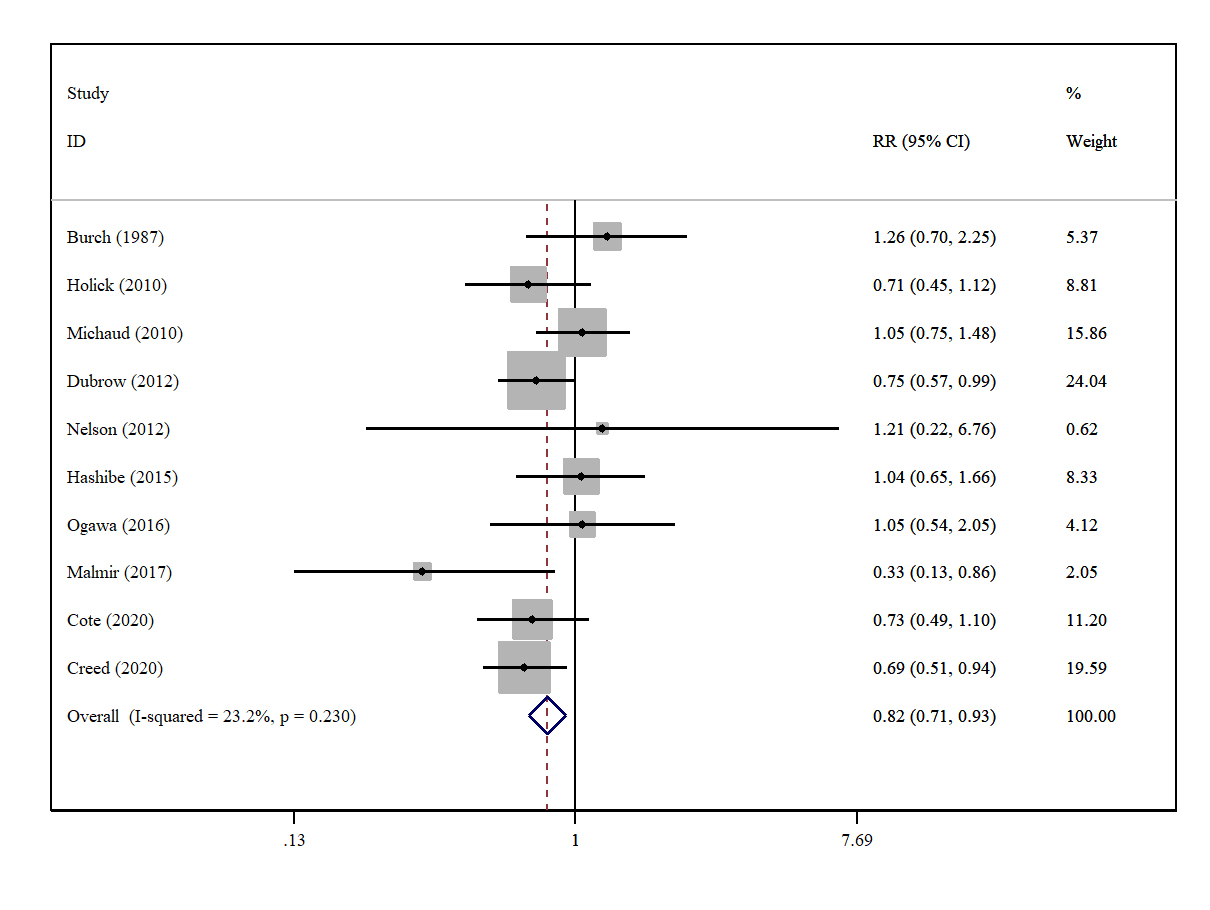
Supplementary Figure 1 | A forest plot showing risk estimates of the association between tea intakes and glioma.


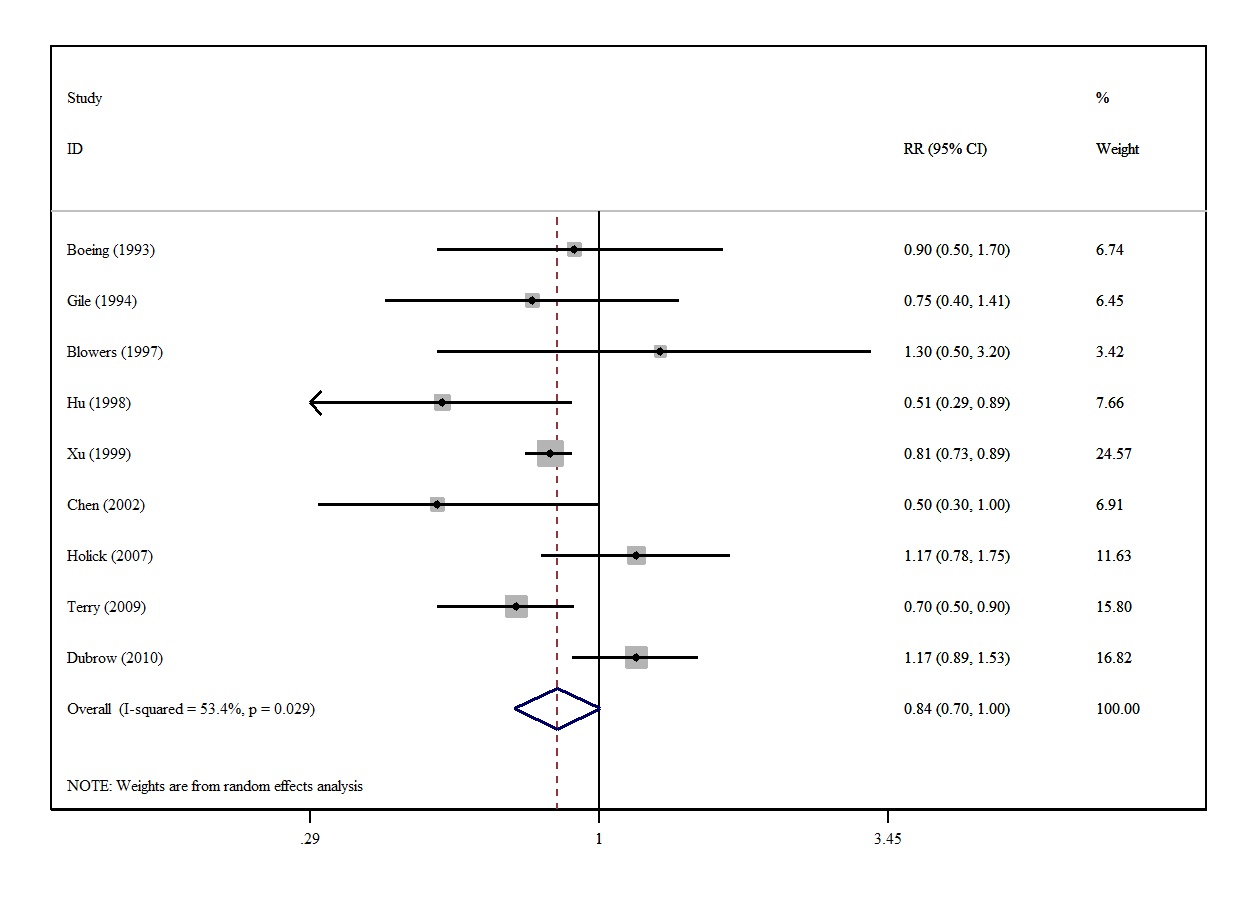
**Supplementary Figure 2 |** A forest plot showing risk estimates of the association between total vegetables intakes and glioma.


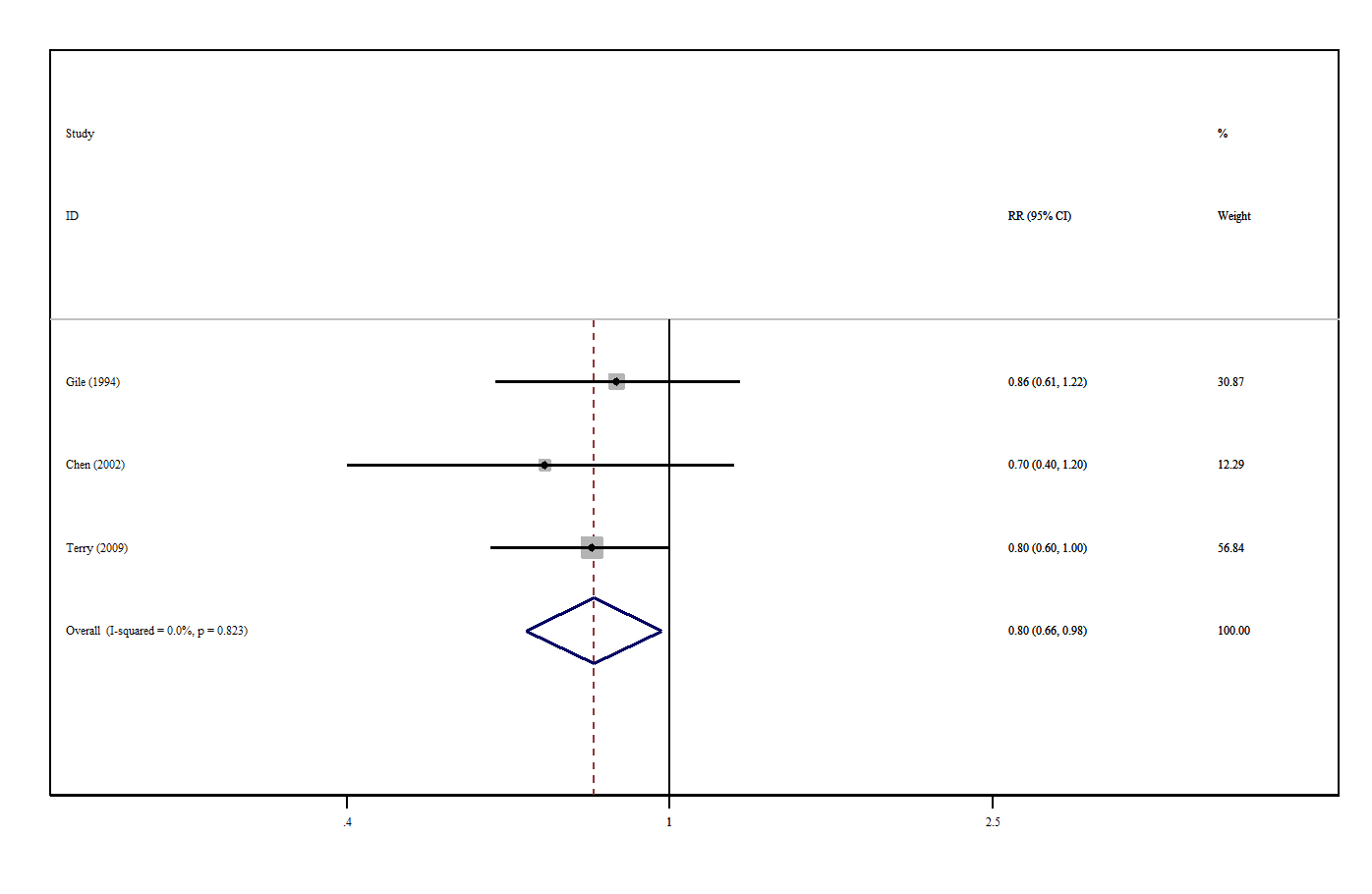


**Supplementary Figure 3 |** A forest plot showing risk estimates of the association between green vegetables intakes and glioma.


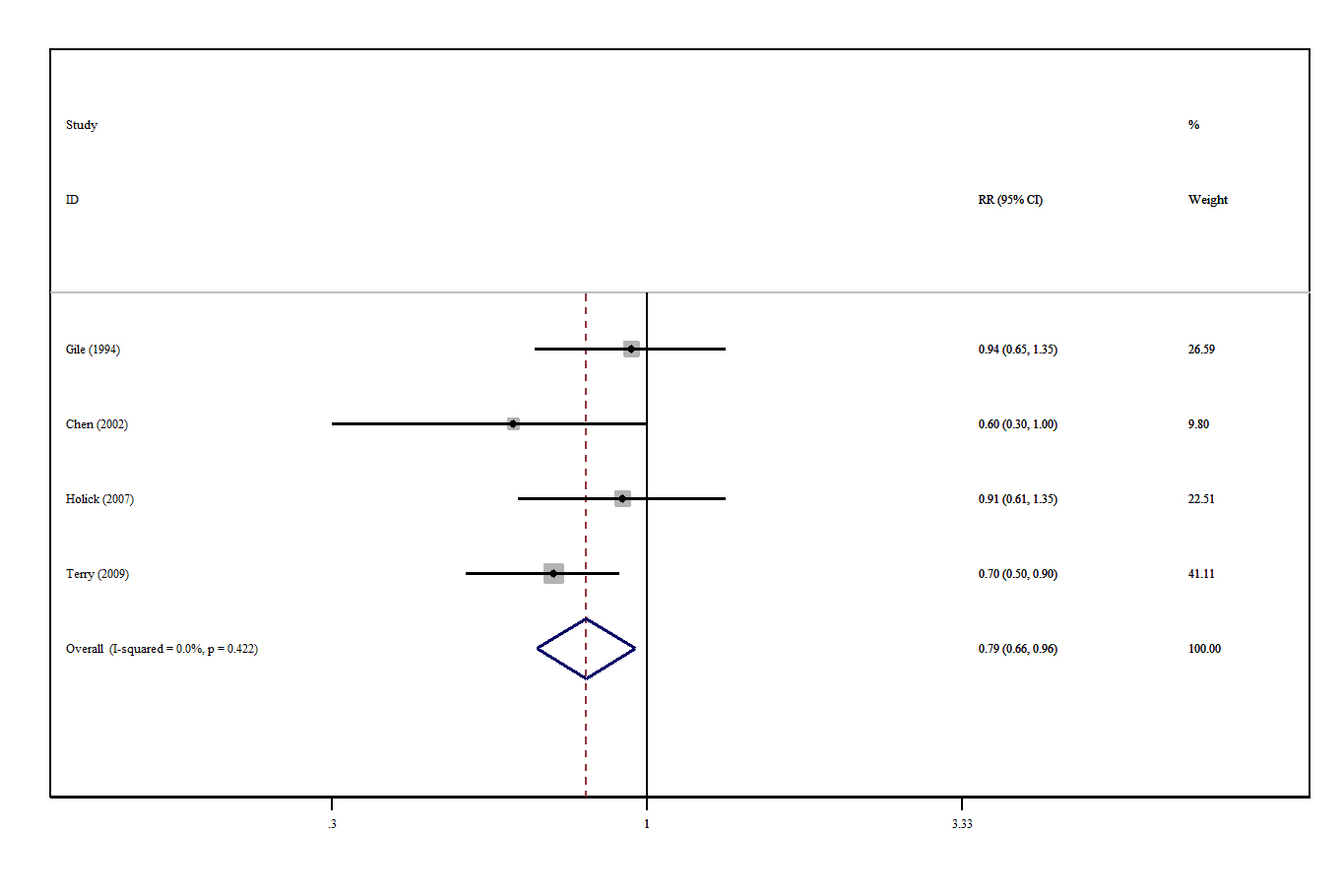


**Supplementary Figure 4 |** A forest plot showing risk estimates of the association between orange vegetables intakes and glioma.


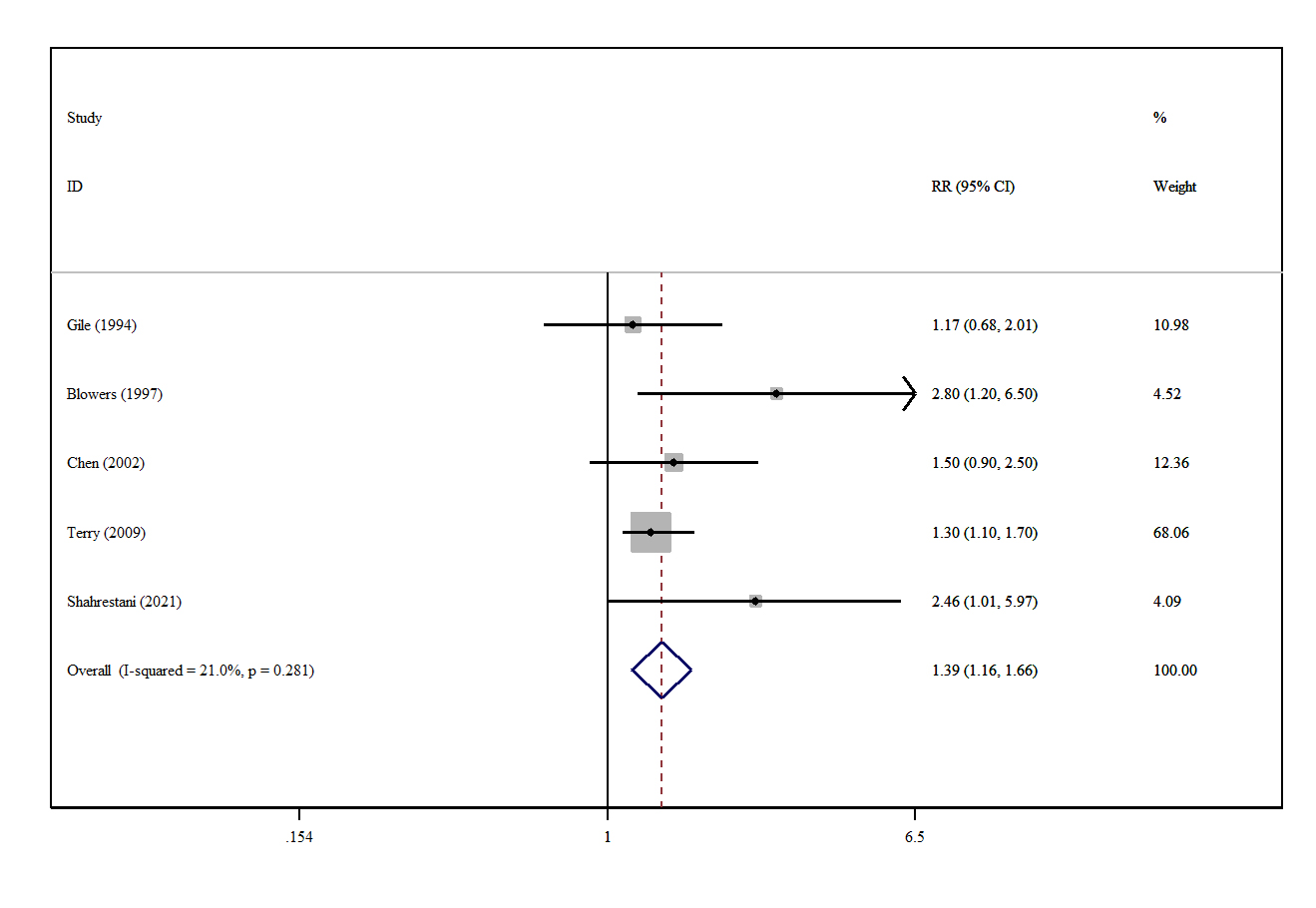


**Supplementary Figure 5 |** A forest plot showing risk estimates of the association between grains intakes and glioma.


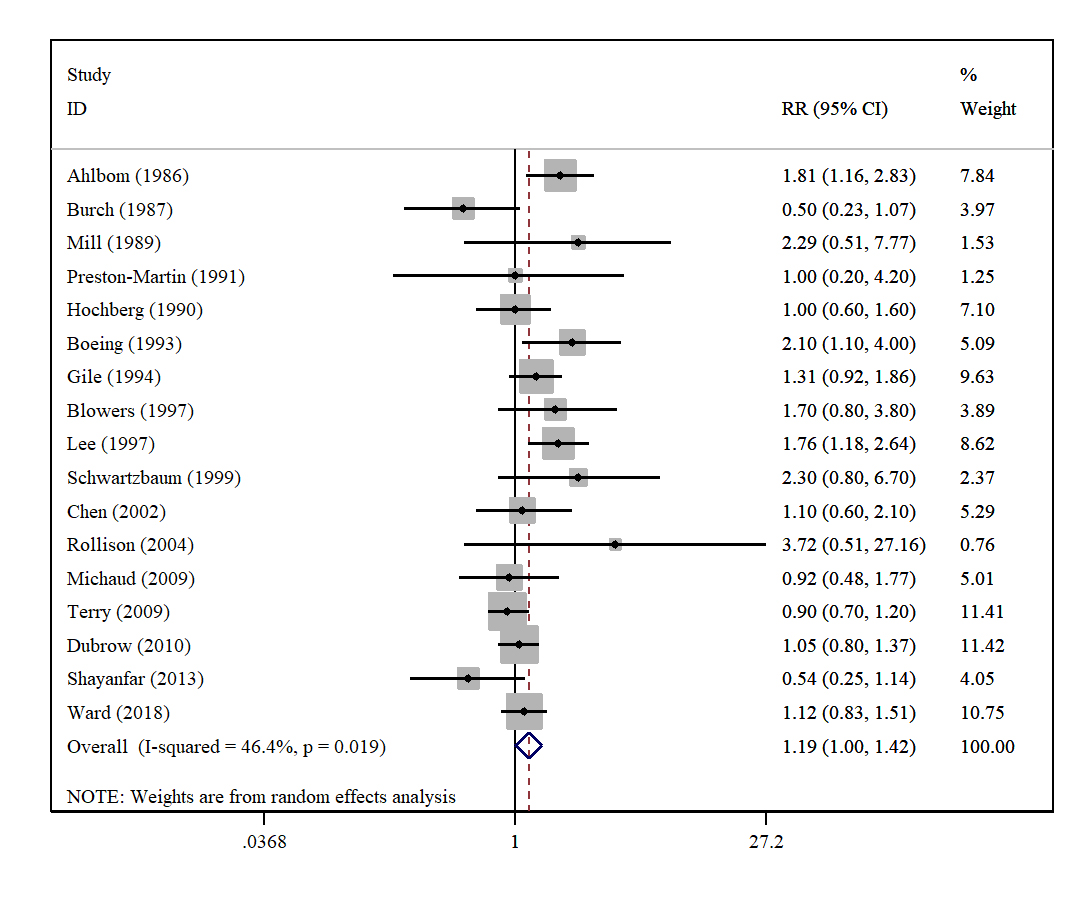


**Supplementary Figure 6 |** A forest plot showing risk estimates of the association between processed meats intakes and glioma.


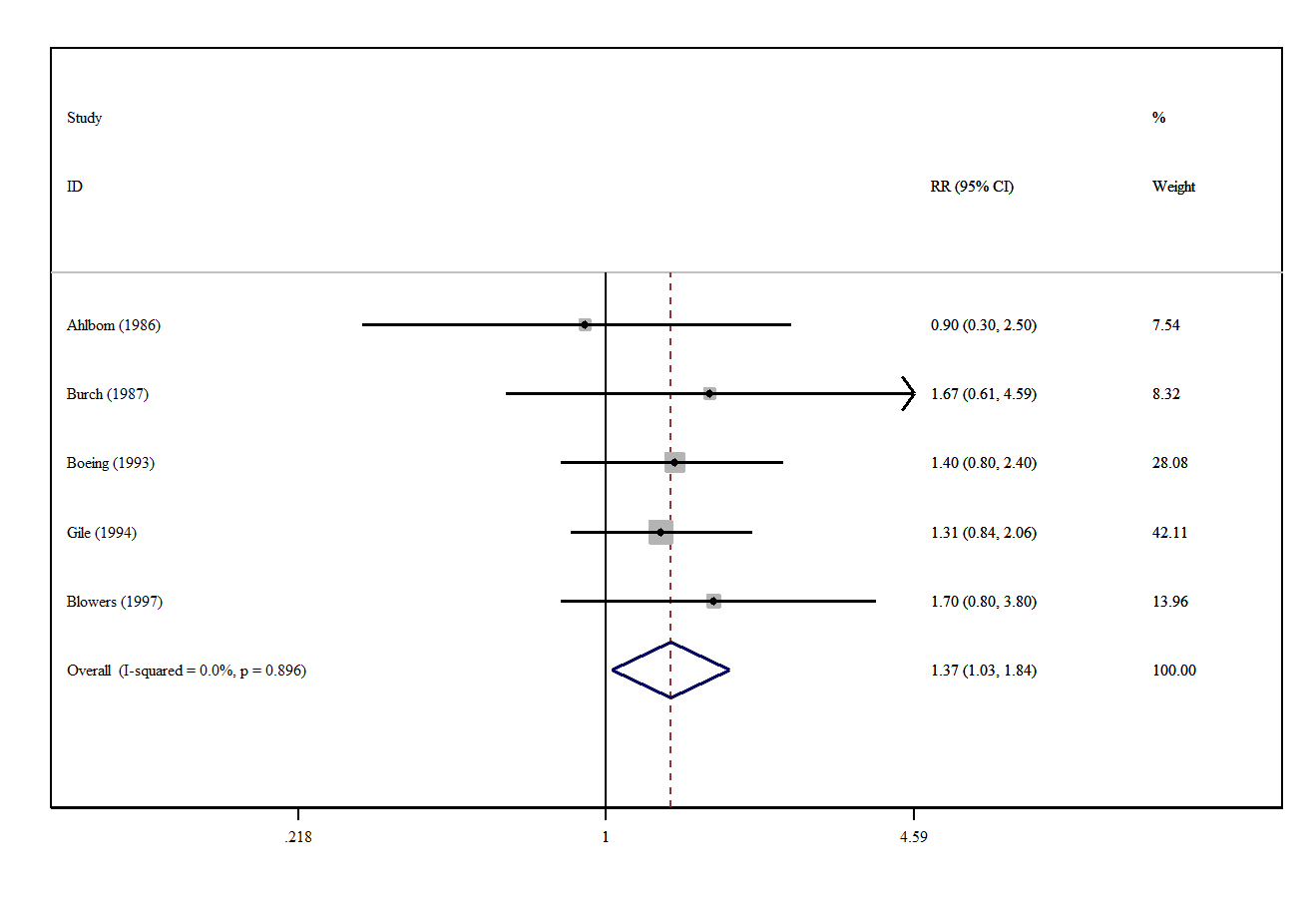


**Supplementary Figure 7 |** A forest plot showing risk estimates of the association between processed fish intakes and glioma.


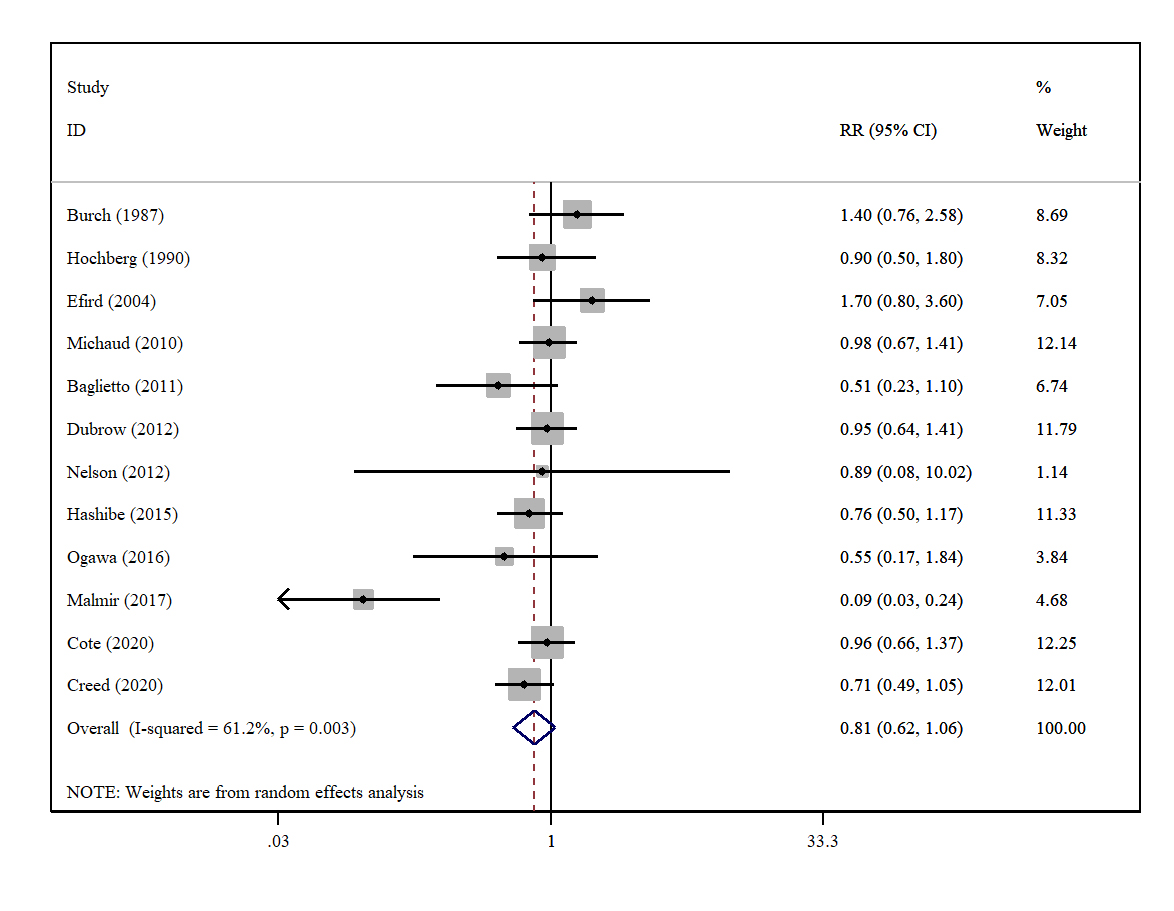


**Supplementary Figure 8 |** A forest plot showing risk estimates of the association between coffee intakes and glioma.


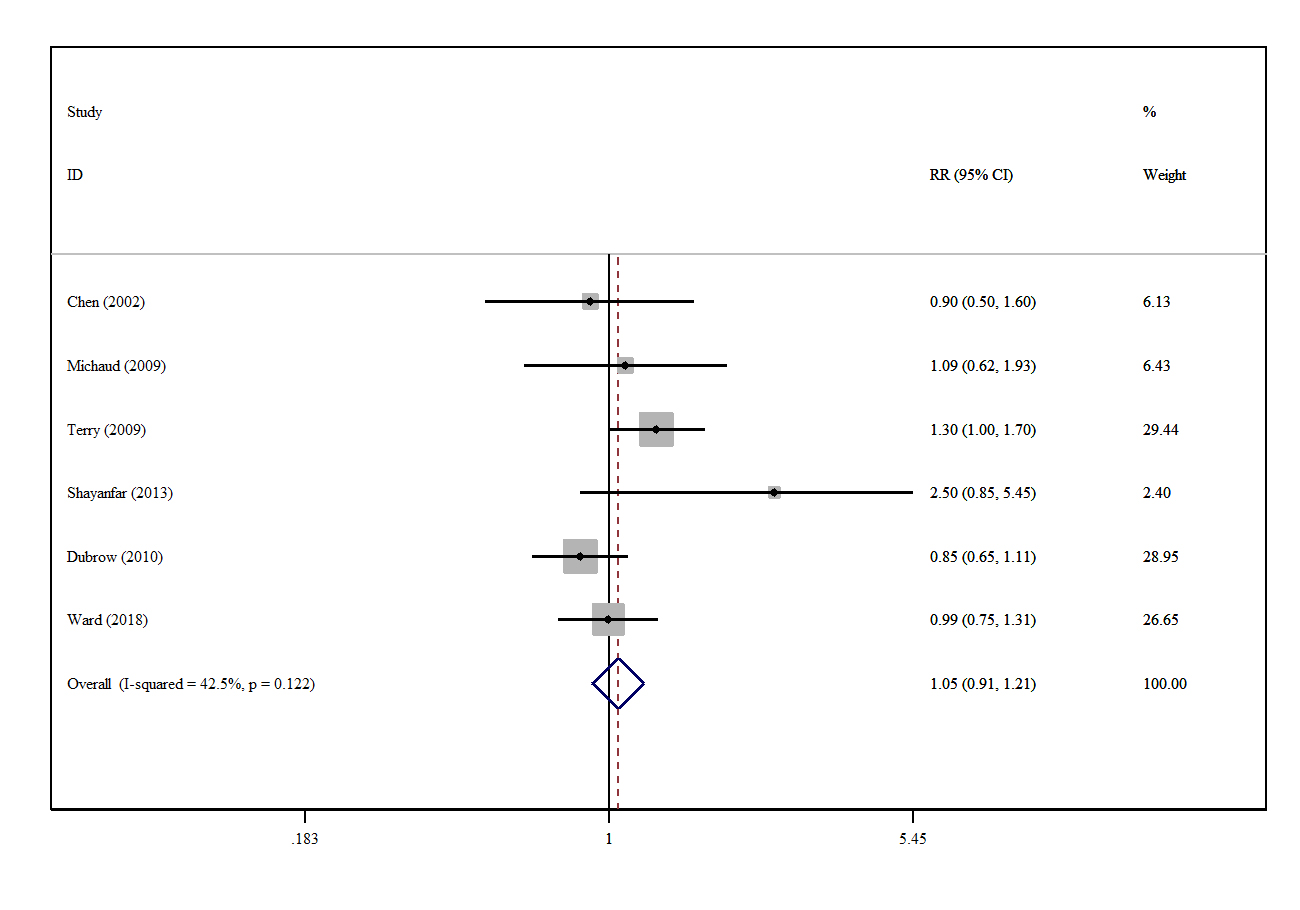


**Supplementary Figure 9 |** A forest plot showing risk estimates of the association between red meats intakes and glioma.


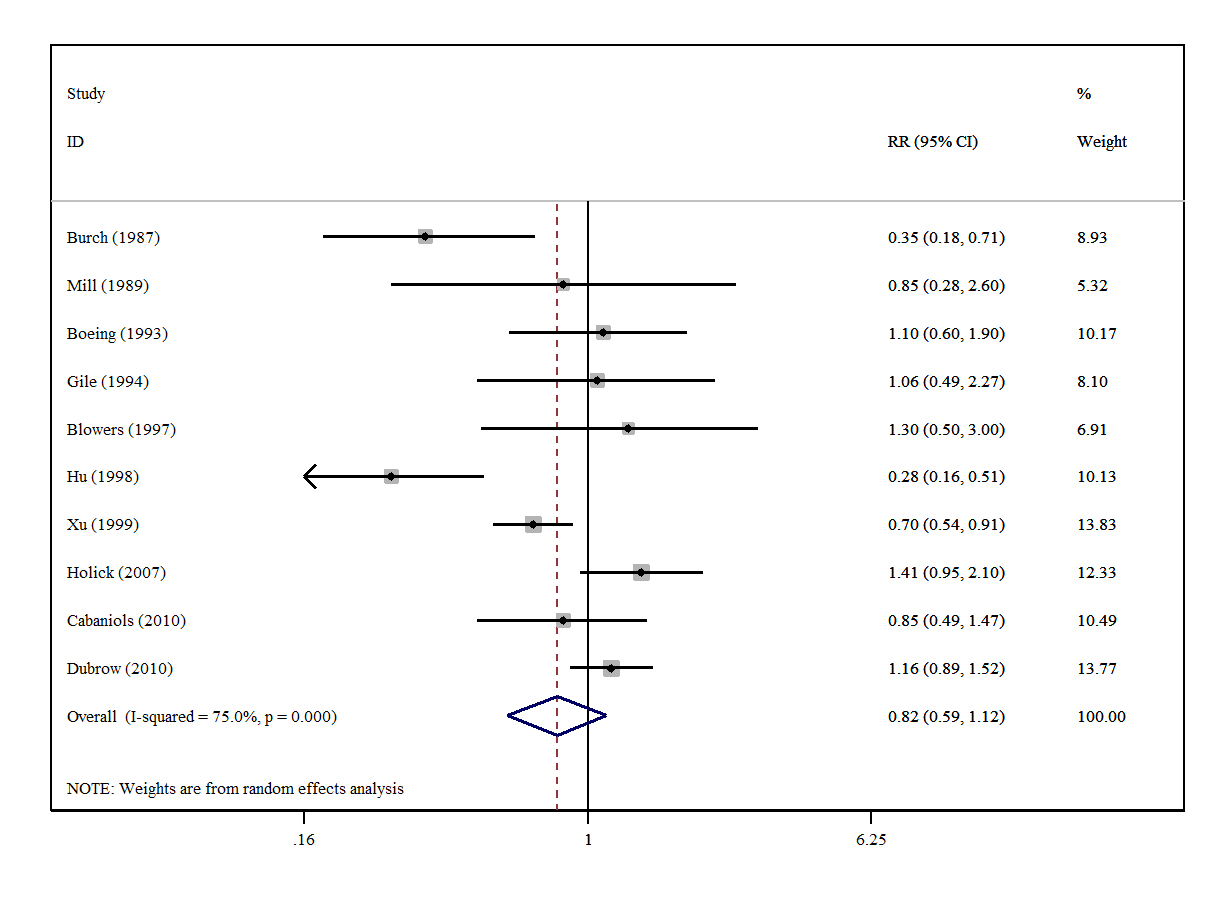


**Supplementary Figure 10 |** A forest plot showing risk estimates of the association between total fruits intakes and glioma.


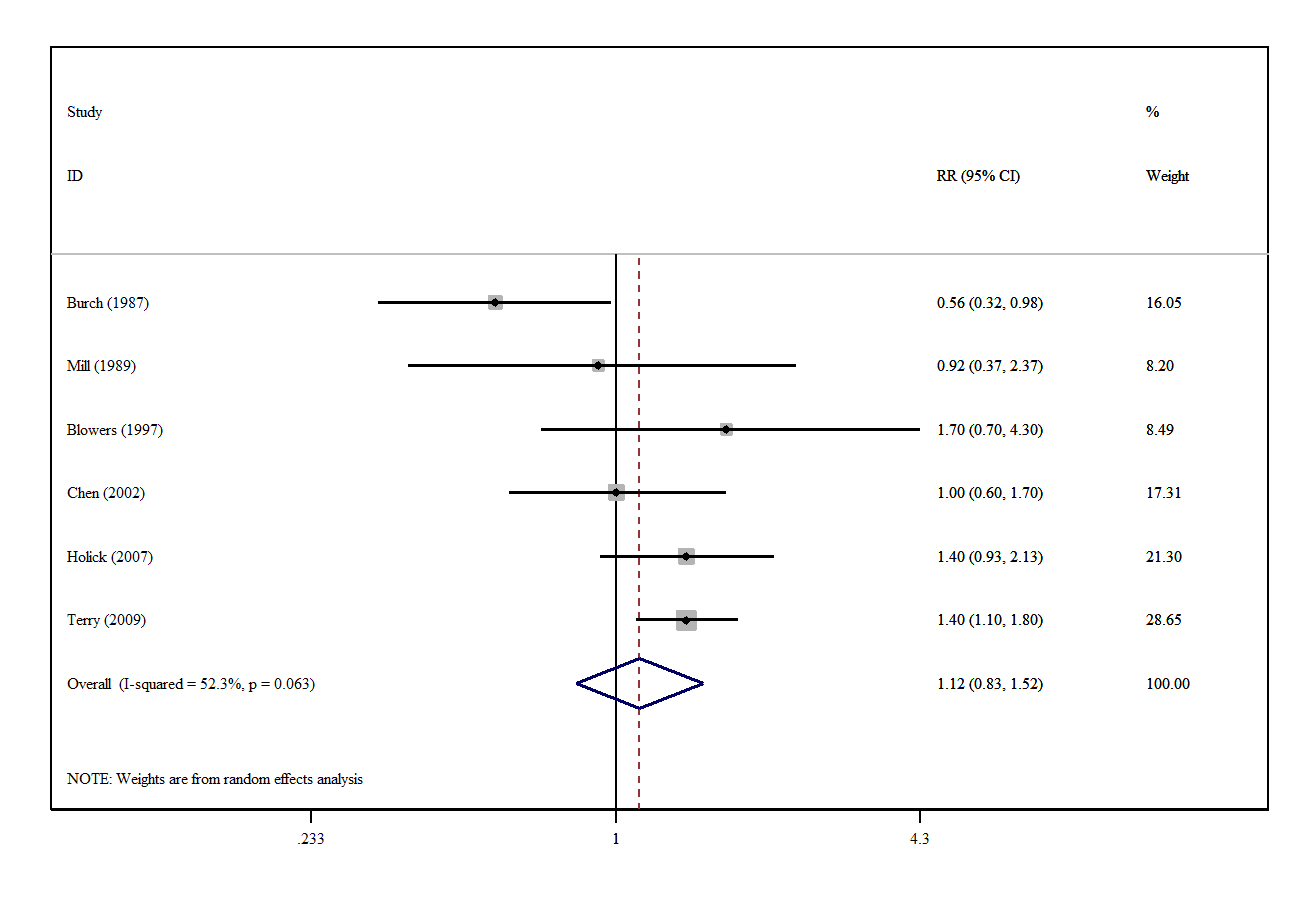


**Supplementary Figure 11 |** A forest plot showing risk estimates of the association between citrus fruits intakes and glioma.


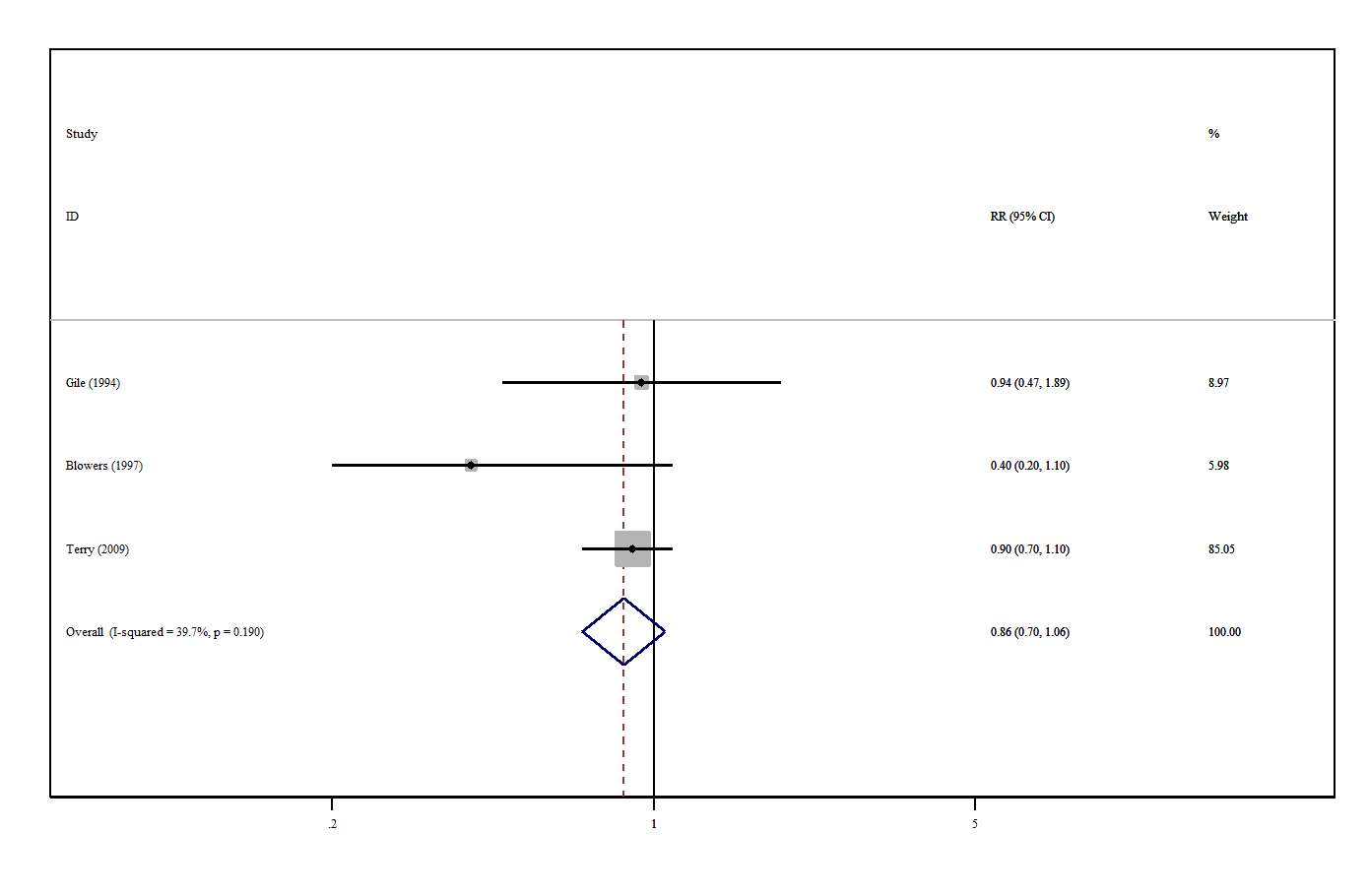


**Supplementary Figure 12 |** A forest plot showing risk estimates of the association between fresh fish intakes and glioma.
